# DanMAC5: a browser of aggregated sequence variants from 8,671 whole genome sequenced Danish individuals

**DOI:** 10.1186/s12863-023-01132-7

**Published:** 2023-05-27

**Authors:** Karina Banasik, Peter L. Møller, Tanya R. Techlo, Peter C. Holm, G. Bragi Walters, Andrés Ingason, Anders Rosengren, Palle D. Rohde, Lisette J. A. Kogelman, David Westergaard, Troels Siggaard, Piotr J. Chmura, Mona A. Chalmer, Ólafur Þ. Magnússon, Guðmundur Á. Þórisson, Hreinn Stefánsson, Daníel F. Guðbjartsson, Kári Stefánsson, Jes Olesen, Simon Winther, Morten Bøttcher, Søren Brunak, Thomas Werge, Mette Nyegaard, Thomas F. Hansen

**Affiliations:** 1 Translational Disease Systems Biology, Novo Nordisk Foundation Center for Protein Research, Faculty of Health and Medical Sciences, University of Copenhagen, Blegdamsvej 3B, DK-2200 Copenhagen N, Denmark; 2 Department of Biomedicine, Aarhus University, Høegh-Guldbergsgade 10, DK-8000 Aarhus C, Denmark; 3 Danish Headache Center, Department of Neurology, Copenhagen University Hospital, Valdemar Hansensvej 1-13, DK-2600 Glostrup, Denmark; 4 deCODE genetics, Sturlugata 8, IS-101 Reykjavik, Iceland; 5 Institute for Biological Psychiatry, Mental Health Center Sct Hans, Copenhagen University Hospital, Boeserup vej 2, DK-4000 Roskilde, Denmark; 6 Present Address: Department of Health Science and Technology, Genomic Medicine Group, Aalborg University, Selma Lagerløfs Vej 249, DK-9260 Gistrup, Denmark; 7 Department of Cardiology, University Clinic for Cardiovascular Research, Gødstrup Hospital, Hospitalsvej 15, DK-7400 Herning, Denmark

**Keywords:** Whole genome sequencing, Variant interpretation, Sequence variants, Minor allele counts, Browser

## Abstract

**Objectives:**

Allele counts of sequence variants obtained by whole genome sequencing (WGS) often play a central role in interpreting the results of genetic and genomic research. However, such variant counts are not readily available for individuals in the Danish population. Here, we present a dataset with allele counts for sequence variants (single nucleotide variants (SNVs) and indels) identified from WGS of 8,671 (5,418 females) individuals from the Danish population. The data resource is based on WGS data from three independent research projects aimed at assessing genetic risk factors for cardiovascular, psychiatric, and headache disorders. To enable the sharing of information on sequence variation in Danish individuals, we created summarized statistics on allele counts from anonymized data and made them available through the European Genome-phenome Archive (EGA, https://identifiers.org/ega.dataset:EGAD00001009756) and in a dedicated browser, DanMAC5 (available at www.danmac5.dk). The summary level data and the DanMAC5 browser provide insight into the allelic spectrum of sequence variants segregating in the Danish population, which is important in variant interpretation.

**Data description:**

Three WGS datasets with an average coverage of 30x were processed independently using the same quality control pipeline. Subsequently, we summarized, filtered, and merged allele counts to create a high-quality summary level dataset of sequence variants.

## Objective

WGS is becoming increasingly accessible and cost-efficient in both basic and clinical research. Thus, it is relevant to be able to assess whether a given variant exists or whether a given genomic region is constrained or not. Because sequence variants are correlated with geographical location, it is of fundamental importance to have variant counts from different countries and regions when linking phenotype to genotype and many large-scale sequencing studies are for this reason making allele counts available to the research community in an anonymised form (gnomAD etc.). In Denmark, several large studies using genotyping arrays exist, e.g., [1–3], however, few sequencing projects have been conducted and none of them have made the allele counts readily available to the research community. Here, we present DanMAC5, allele counts for sequence variants from 8,671 Danish individuals identified through WGS of three independent studies made available through the accompanying DanMAC5 browser and via EGA. To protect participant privacy and enable a joint data resource, all allele counts below five have been masked and is displayed as < 5. The DanMAC5 dataset and browser represents an important open resource of observed single nucleotide variant (SNV) and indel allele counts segregating in the Danish population and can be used for sequence variant filtering in the wider genetics and genomics research community.

## Data description

### Demographics

Data from three studies were included:

- Dan-NICAD: 1,649 individuals with symptoms of obstructive coronary artery disease, predominantly chest pain, undergoing coronary computed tomography angiography. In total, 52% were females, the mean age was 57 years (+/- 9 SD), median coronary artery calcium score were 0 [0–82] and 24% of the cohort had obstructive coronary artery disease defined as > 50 diameter stenosis at angiography [4–6].
- IBP: Historical data from 3,675 (2,155 females) irrevocably anonymized samples originally collected at the then H:S Sct. Hans Hospital.
- Migraine: 3,347 (2,406 females) patients from the Danish Headache Center, including families with clustering of migraine [7, 8].

Permissions for the included studies were obtained from the Danish Data Protection Agency and the appropriate Scientific Ethical Committee system.

### Whole genome sequencing

WGS data was generated in three independent research projects [4, 7, 9]. In short, genomic DNA was isolated from frozen whole-blood in EDTA tubes with no DNA amplification or enrichment. Sequencing libraries were prepared using TruSeq PCR-Free (Illumina) and sequenced on the Illumina sequencing platform (NovaSeq 6000 or HiSeq) with S4 flow cells using 2 × 150 bp paired end sequencing. WGS data underwent quality control using the in-house pipeline at deCODE genetics that has been described previously [10, 11]. Genotype calls were generated per individual with GATK HaplotypeCaller v4.3.3 [12]. The VCF-formatted result files were merged, filtered and aggregate counts generated using bcftools v1.14 [13]. The filtering step was performed as follows: variants with a QUAL-score (QD) < 2.0, Root Mean Square of the mapping quality (MQ) < 40.0, and strand bias by Fisher exact (FS) > 60 were excluded [10]. Anonymized allele counts from each research project were annotated to the GRCh38 version of the human genome (GCA_000001405.15_GRCh38_no_alt_analysis_set.fna [14]) were subsequently merged.

An additional extended quality control was performed by removing low-quality variants using a “whitelist” which was based on a rigid variant calling in two cohorts, Dan-NICAD [4, 5] and migraine [7]; base quality score recalibration (BQSR) was performed using recalibration tables generated with the Sentieon QualCal algorithm. GVCFs were created for each individual using the Haplotyper algorithm before merging with GVCFtyper [15]. Variant quality score recalibration (VQSR) was performed independently for SNPs and indels, based on hapmap3, 1000 genomes, and dbSNP resources, using a sensitivity threshold of 99.7 for passing variants.

After merging and additional quality control filtering using the whitelist, variants with minor allele counts (MAC) of less than five (i.e., seen one to four times) were reported as “<5” to ensure participants’ privacy. Sequence variants on the Y chromosome and mitochondria are not reported. A total of 8,671 samples passed the standard quality measures, with an average coverage of 30 reads.

### Browser

Using the Dash web-framework (https://plotly.com/dash/) we created an interactive data browser which is available at www.danmac5.dk. Queries can be made using rsID, variant position (chr:pos), gene name (RefSeq), or genomic ranges (chr:pos-pos). All positions are GRCh38/hg38. A hyperlink to gnomAD [16] v3.1.2 (based on hg38) is available in the rsID column. Table 1 lists the file that hold DanMAC5 data and where the features of the DanMAC5 browser are extracted from.

**Table 1:** Overview of data files/datasets

Label	Name of data file	File types (file extension)	Data repository and identifier
*Data file 1*	*all_mac5*	*Variant Call Format (.vcf)*	*European Genome-phenome Archive* https://identifiers.org/ega.dataset:EGAD00001009756

## Limitations

- Sequence variants cannot be linked to the individual’s disease status.
- Our sample contains related individuals which may result in slightly over- or underestimated allele counts.
- Variants with a total allele count below five are listed as < 5 to enable the sharing of data for population genetics and protect the privacy of participants.
- Larger structural variants, variants on the Y chromosome, and mitochondrial variants were not assessed.
- Genomic regions containing repetitive sequences could not be retrieved using pair-end sequencing.

## Data Availability

The DanMAC5 data described in this Data note can be freely and openly accessed on www.danmac5.dk. Please see references [4, 7–9] for details and links to the original studies. **Download** The full dataset is available for academic use for bona fide researchers via https://identifiers.org/ega.dataset:EGAD00001009756 upon registration via the European Genome-phenome Archive: providing clear terms and conditions for use of the full dataset [17]. Please refer to the European Genome-phenome Archive (https://ega-archive.org/access/data-access) for details on how to register.

## Abbreviations

Dan-NICAD: Danish study of Non-Invasive testing in Coronary Artery Disease
EGA: European Genome-phenome Archive
GVCF: genomic variant call format
IBP: Institut for Biologisk Psykiatri (Research Institute of Biological Psychiatry)
MAC: minor allele counts
SNV: single nucleotide variant
WGS: whole-genome sequencing
